# Editorial: Increasing production of crops with bioavailable micronutrients: a solution to reduce global malnutrition

**DOI:** 10.3389/fpls.2023.1348246

**Published:** 2023-12-15

**Authors:** Faisal Nadeem, Abdul Rehman, Spyridon A. Petropoulos

**Affiliations:** ^1^Department of Agronomy, University of Agriculture, Dera Ismail Khan, Khyber Pakhtunkhwa, Pakistan; ^2^Department of Agronomy, Faculty of Agriculture and Environment, The Islamia University of Bahawalpur, Bahawalpur, Pakistan; ^3^Laboratory of Vegetable Production, Department of Agriculture, Crop Production and Rural Environment, University of Thessaly, Volos, Greece

**Keywords:** hidden hunger, biofortification, bioavailability, food security, nutritional quality

Malnutrition refers to diets that do not contain the proper amounts of nutrients, while hidden hunger refers to micronutrient deficiencies and affects almost one third of world population (Dwivedi et al., 2023). Recent scientific advances have focused on biotechnology and agronomic practices that may improve the nutritional quality of crops and contribute to combating malnutrition and ensure food security (Kumar et al., 2023; Singh Dhaliwal et al., 2023; Singh et al., 2023).

This Research Topic compiles innovative research studies and review reports that gather up the most up-to-date information regarding the use of agronomic techniques that can be applied to various crops and result in higher bioavailability of micronutrients without compromising yield and quality of the final product.

Yang et al. investigated the selenium (Se) uptake and distribution dynamics in rice plants via Algal Polysaccharides–Selenium Nanoparticles (APS-SeNPs). Their hydroponic study, anchored in the Michaelis–Menten equation, revealed a notable Vmax of 13.54 μg g^−1^ root dry weight/hour for APS-SeNPs — 7.69 and 2.23 times higher than selenite and selenate treatments, respectively. Silver nitrate (AgNO_3_) and carbonyl cyanide 3-chlorophenylhydrazone (CCCP), inhibited the root uptake of APS-SeNP by 64.81% to 79.09% and 19.83% to 29.03%, respectively which indicated aquaporins play an essential role in the primary uptake of APS-SeNP in rice. Furthermore, this study underscored APS-SeNPs efficacy in enhancing Se uptake and defining its unique distribution patterns in rice

Xue et al. explored maize biofortification through foliage fertilizer applications including ZnO nanoparticles, Zn complexed chitosan nanoparticles (Zn-CNPs), conventional ZnSO_4_, and a comprehensive cocktail solution (Zn, Fe, Se) across three maize cultivars grown in three different locations. The application of conventional ZnSO_4_ efficiently increased grain Zn concentration, while a minimal increase was recorded with reduced rate of ZnO-NPs, Zn-CNPs, or ZnSO_4_. Notably, the cultivar with the lowest yield exhibited the most pronounced increase in Zn and Fe. The cocktail solution also reduced phytic acid concentrations, enhancing Fe and Zn bioavailability, emphasizing the need for a delicate balance between achieving high grain yield and maintaining optimal nutritional quality in effective biofortification.

Chen et al. presented a comprehensive meta-analysis, a meticulous examination of 1,193 data records from 38 publications, providing a nuanced understanding of the intricate relationship between selenium (Se) and mercury (Hg) accumulation in plants. Their findings revealed a dose-dependent reduction in Hg concentration, with an optimal Se/Hg ratio of 1–3, highlighting the efficacy of Se in mitigating Hg accumulation in various plant species, particularly in rice grains. This discovery suggests a promising strategy to curtail Hg transfer through the food chain, underscoring the potential of Se supplementation in reducing environmental toxicological risks.

Magor et al. delved into the pressing issue of iodine deficiency, investigating foliar application strategies for iodine fortification in wheat. Their glasshouse experiment, incorporating nine treatments with various adjuvants and controls, demonstrated the significant impact of the organosilicon-based adjuvant (Pulse®). This addition notably enhanced grain iodine loading to 1269 µg/kg, compared to the non-adjuvant KIO_3_ control at 231 µg/kg. Additionally, Synerterol® Horti Oil emerged as the second most effective adjuvant, elevating grain iodine to 450 µg/kg. Importantly, the study showed that iodine application did not compromise biomass or grain yield, underlining the potential of using adjuvants, especially organosilicon-based ones, to effectively enhance iodine biofortification in staple grains. Both these studies contributed vital knowledge to the pursuit of sustainable approaches for reducing global malnutrition by optimizing crop micronutrient content.

Kathi et al. evaluated the potential of biofortifying broccoli microgreens with vitamin C by adding increments of ascorbic acid (0% to 0.5%) in nutrient solution. The results of this study suggested that the addition of ascorbic acid not only increased vitamin C (total ascorbic acid and ascorbic acid) content of microgreens but also affected yield, dry matter and chlorophylls, carotenoids and minerals content in a dose dependent manner.

On the other hand, Sánchez-Palacios et al. performed a literature review regarding the biofortification of wheat through foliar application of Zn, focusing on Zn assimilation by wheat plants and its translocation to grain and consequently to biofortified wheat bread. Moreover, in the same study the efficacy of adjuvants and novel nano-transporters compared to conventional Zn forms was discussed, suggesting that novel agronomic practices may allow wheat cultivation in Zn-deficient soils while improving Zn content in wheat grains, thus contributing in malnutrition mitigation.

Finally, Wang et al. performed a combined analysis of inorganic elements and flavonoids aiming to reveal the relationship of quality parameters and maturity stage of dried flower buds of Sophora japonica L. The obtained results suggested a variable pattern of minerals and flavonoids accumulation at different maturity stages, while the authors reported that the content of specific minerals such as Ca, P, K, Fe, and Cu may affect the biosynthesis of flavonoid metabolites.

In conclusion, the studies compiled in this Research Topic contributed vital knowledge to the pursuit of sustainable approaches for reducing global malnutrition by optimizing crop micronutrient content.

## Author contributions

SP: Writing – original draft, Writing – review & editing. FN: Writing – original draft, Writing – review & editing. AR: Writing – original draft, Writing – review & editing.
